# Medical students value advocacy and health policy training in undergraduate medical education: A mixed methods study

**DOI:** 10.1017/cts.2025.35

**Published:** 2025-02-28

**Authors:** Caroline Minnick, Kevin Alexander Soltany, Sudarshan Krishnamurthy, Maeve Murray, Roy Strowd, Kimberly Montez

**Affiliations:** 1 Bowman Gray Center for Medical Education, Wake Forest University School of Medicine, Winston-Salem, NC, USA; 2 Department of Neurology, Wake Forest University School of Medicine, Winston-Salem, NC, USA; 3 Department of Pediatrics, Wake Forest University School of Medicine, Winston-Salem, NC, USA; 4 Department of Social Sciences and Health Policy, Division of Public Health Sciences, Wake Forest University School of Medicine, Winston-Salem, NC, USA

**Keywords:** Social determinants of health, medical education, advocacy, health policy, qualitative, interview, survey, curriculum

## Abstract

**Introduction:**

This study aimed to describe medical students’ perceptions and experiences with health policy and advocacy training and practice and define motivations and barriers for engagement.

**Methods:**

This was a mixed-methods study of medical students from May to October 2022. Students were invited to participate in a web-based survey and optional follow-up phone interview. Surveys were analyzed using descriptive statistics. Phone interviews were audio-recorded, transcribed, and de-identified. Interviews were coded inductively using a coding dictionary. Themes were identified using thematic analysis.

**Results:**

35/580 survey responses (6% response rate) and 15 interviews were completed. 100% rated social factors as related to overall health. 65.7% of participants felt “very confident” or “extremely confident” in identifying social needs but only 11.4% felt “very confident” in addressing these needs. From interviews, six themes were identified: (1) participants recognized that involvement in health policy and/or advocacy is a duty of physicians; (2) participants acknowledged physicians’ voices as well respected; (3) participants were comfortable identifying social determinants of health but felt unprepared to address needs; (4) barriers to future involvement included intimidation, self-doubt, and skepticism of impact; (5) past exposures and awareness of advocacy topics motivated participants to engage in health policy and/or advocacy during medical school; and (6) participants identified areas where the training on these topics excelled and offered recommendations for improvement, including simulation, earlier integration, and teaching on health-related laws and policies.

**Conclusions:**

This study highlights the importance of involvement in health policy and advocacy among medical students and the need for enhanced education and exposure.

## Introduction

Following the 2020 surge of a cultural movement in the United States (US) addressing racial inequities, the perception of healthcare professionals as social advocates has significantly intensified [1,2]. This focus on healthcare advocacy builds on a long-standing tradition of physician advocacy, with roots extending centuries back. Prominent examples include Dr. Rudolf Virchow, the “father of modern pathology,” who declared medicine a “social science” in his 1848 report on the typhus epidemic, emphasizing the influence of poverty, famine, and corruption on health [3]. In recent decades, advocacy has been formally recognized by major organizations like the American Board of Internal Medicine and the American Medical Association, which incorporated advocacy into their mission statements as early as 2002. [4–6] Individuals in medicine, both practitioners and students, now are keenly attuned to the pervasive racial, ethnic, and social injustices within the field. Medical literature, news outlets, and opinion pieces are replete with calls to action for physicians, urging them to leverage their expertise in and understanding of the profound impact of social determinants of health (SDOH) to address inequities within our healthcare system [7]. As racial, ethnic, and social justice, including health equity, take center stage in the public discourse, an increasing number of physicians are actively participating in these crucial efforts.

The moral obligation of physicians to speak and act against situations of injustice demands that physicians are equipped with adequate skills to effectively advocate for the needs of their communities. Health systems science education, considered by many as the third pillar of medical education, alongside basic and clinical science, encompasses key areas like SDOH, healthcare policy, and advocacy [6,8,9]. The positive impact of such training is described in literature, with notable improvements in students’ understanding of the SDOH and confidence in acting as advocates [10–14]. In addition, foundational knowledge of the US healthcare system is imperative to well-informed and effective advocacy, highlighting the need for paired health policy and advocacy training in undergraduate medical education (UME) [15,16]. Many institutions have begun sharing the design of curricula related to health policy and/or advocacy, and surveys of medical students’ perceptions of these curricula have been disseminated [12,17,18]. These various studies, when considered together, reveal significant gaps in health policy and advocacy training among US medical students.

While previous literature has assessed medical students’ attitudes about advocacy quantitatively, there remains a gap in the literature exploring students’ perceptions and experiences with health policy and advocacy training and practice, and the motivations and barriers for engagement [19]. While there is growing consensus that health policy and advocacy are essential to the physician’s societal role, many studies have shown that few have translated this belief into action through consistent voting, monetary support of candidates, or physical advocacy on the local or national level [1,19–21]. Without documented support for mandatory curricula and exposure within UME, it is challenging to fully endorse programing and develop curricula that attract student engagement. A better understanding of students’ experiences and reasons for pursuing training in health policy and advocacy efforts will inform future programmatic and curricular development. Conversely, identifying potential barriers may reveal awareness gaps, barriers, and biases that could be addressed via training and educational programing to enhance voluntary engagement. This study aimed to explore medical students’ perceptions and experiences with health policy and advocacy training and practice and the motivations and barriers for engagement in these areas.

## Methods

### Study participants and data collection

This was a mixed-methods investigation of Doctor of Medicine (MD) degree-seeking students enrolled at the Wake Forest University School of Medicine (WFUSM), which is located in Northwest North Carolina. At the time of recruitment, 32% of the WFUSM M.D.-degree-seeking student body comprised individuals self-identifying as underrepresented in medicine (i.e., American Indian/Alaska Native, Black/African American, Hispanic, or Native Hawaiian/Pacific Islander) or socioeconomically disadvantaged. Each year, WFUSM accepts 145 students for its incoming first-year class, yielding a student body of approximately 580 students. Mapping data of the WFUSM MD curriculum collected in preparation for the Liaison Committee on Medical Education reaccreditation in 2024 demonstrates that health policy and advocacy learning objectives are embedded throughout all four years of UME, including core pre-clerkship courses and within hospital-based clerkship courses. The pre-clerkship “Medicine and Patients in Society” course and the longitudinal service-learning “Health Equity Thread” for clerkship students highlight WFUSM’s two focused health policy and advocacy courses, spanning all four years of training. The Health Justice Advocacy Certificate and the Health Equity Certificate are two separate, one-year programs that are also available as optional trainings [22].

The study design is concurrent triangulation in which both quantitative and qualitative data were collected during the same timeframe and given equal weight in the interpretation phase. Any student currently enrolled in the WFUSM was eligible to participate.

**Figure 1.**
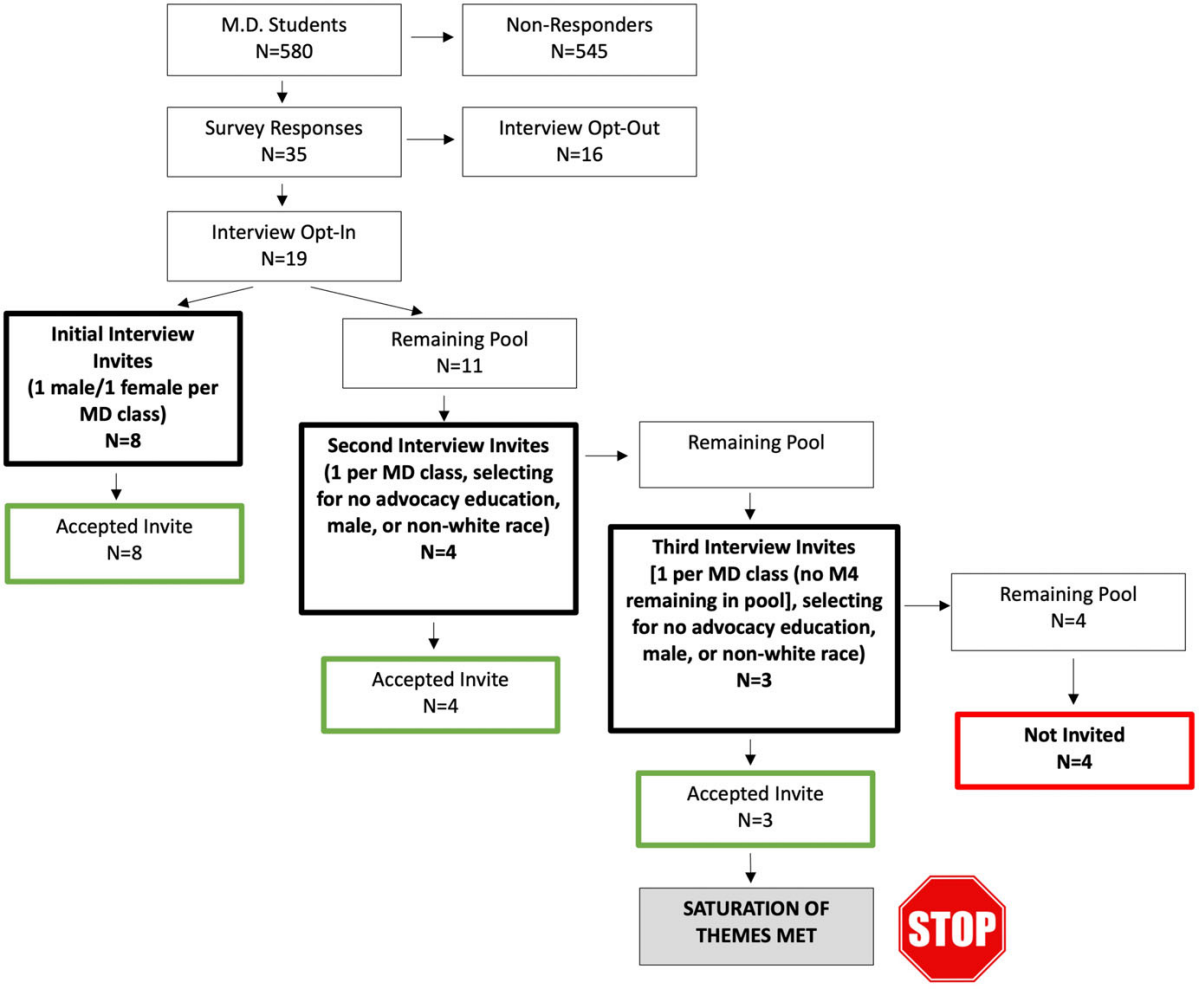
Vertical diagram of interview participant invitation process.

Beginning in May 2022, medical students were invited to participate in our web-based REDCap survey and optional follow-up phone interview. REDCap is a secure, web-based software platform designed to support data capture for research studies [23]. Students were recruited to complete the survey through convenience sampling. The survey link was distributed to all 580 medical students via email, class group messenger, and paper advertisements at the medical school campus. Recruitment continued for a 6-month period. Participants consented to participation via the online survey and self-screened into the demographic portion of the survey, filtering for students actively enrolled in the spring of 2022. At the close of the web-based survey, participants could denote their interest in the phone interview portion of the study. Interview participants were purposively sampled by the study team to ensure diverse representation by gender, year, race, ethnicity, and advocacy experience, reflecting the WFUSM student body (Fig. 1).

Through a detailed review of the literature, we designed an interview guide with an aim of exploring students’ perceptions and experiences with health policy and advocacy training and practice, and the motivations and barriers for engagement [24–26]. The interview guide was conceptually based on the Humanistic Theory, which focuses on exploring participant autonomy and free will, aligning with our primary goal of exploring participant motivations [27]. The interview guide was pilot-tested for face validity, and minor wording changes were made to the guide as a result. Interviews were conducted via phone. After obtaining consent, we conducted semi-structured phone interviews utilizing the interview guide between June and October 2022. The interviews were conducted in English by one researcher (CM) who was trained in qualitative interview techniques. This researcher (CM) was also a medical student who had been on the student executive committee of the Health Justice Advocacy Certificate. All the study participants were known to the researcher prior to the interview.

Study participants shared their understanding of the meaning of advocacy, their experiences with and motivations to participate in advocacy and health policy efforts during medical school, their views on how health policy and advocacy will influence their future medical careers, and recommendations around health policy and advocacy curricula. Interviews lasted approximately 30 minutes (range 17–48 minutes). There was no monetary or other form of compensation for participation in either the web-based survey or the phone interview. Interviews were continued until thematic saturation was deemed to have been achieved, defined as the degree to which new data repeated what was expressed in previous data [28]. Each of the phone interviews was audio-recorded, transcribed verbatim, and de-identified to maintain participant confidentiality.

### Data analysis

Data from web-based surveys were collected in REDCap and subsequently exported in raw format. Descriptive statistics were utilized to demonstrate distribution of responses among the survey participants.

Transcribed narrative data were transferred to Atlas.ti (Version 8) Scientific Software Development GmbH (Berlin, Germany) for further analysis and coding. Interviews were coded inductively as codes emerged from the data set. Following coding of the first five interviews, a coding scheme and dictionary were set in as a guide for additional interviews. Each transcript was coded individually by two individuals (CM and MM) who assigned codes to phrases and portions of the transcripts based on the developed scheme. The codes for each interview were compared for consistency. Discrepancies in coding were discussed among the two coders and resolved iteratively. As new codes emerged, the coding scheme and dictionary were updated to reflect consensus among the coding team. Segments of the text were synthesized into themes using the principles of thematic analysis [29]. The WFUSM Institutional Review Board approved this study.

## Results

### Survey participant demographics

A total of 35/580 survey responses were recorded, yielding a response rate of 6%. Most survey participants were non-Hispanic white (21/35, 60%), male (15/35, 57.1%), and were aged 22–25 years (19/35, 54%). Regarding the distribution of survey participants across medical school classes, the majority (11/35, 31.4%) were from the first-year class (M1), followed by M2 (10/35, 28.6%), M3 (9/35, 25.7%), and M4 (5/35, 14.3%) (Table 1).

**Table 1.** Survey and interview participant demographics

	Interview participants (N = 15)	Survey participants (N = 35)
	**% (N)**	**% (N)**
**Age**		
22-25	53.33% (8)	54.29% (19)
26-29	40% (6)	40% (14)
30+	6.67% (1)	5.71% (2)
**Gender**		
Male	40% (6)	57.14% (15)
Female	60% (9)	42.86% (20)
**Race**		
White	60% (10)	77.14% (27)
Black	6.67% (1)	5.71% (2)
Asian	26.67% (3)	14.29% (5)
Mixed Race	6.67% (1)	2.86% (1)
**Ethnicity**		
Non-Hispanic	86.67% (12)	80% (28)
Hispanic	6.67% (2)	17.14% (6)
Other	6.67% (1)	2.86% (1)
**Class/Year**		
M1	26.67% (4)	31.43% (11)
M2	26.67% (4)	28.57% (10)
M3	26.67% (4)	25.71% (9)
M4	20% (3)	14.29% (5)
**Focused advocacy education**		
Yes	73.33% (11)	51.43% (18)
No	26.67% (4)	48.57% (17)

### Interview participant demographics

From the 35 survey participants, 19/35 (54%) expressed interest in participating in the semi-structured phone interviews, and 15 were ultimately selected. Among the interviewees, there was a balanced representation across M1-M4 classes, with 40% (6/15) being male and 53% (8/15) identifying as non-Hispanic white. Notably, 73% (11/15) of interview participants were involved in focused advocacy education, defined as enrollment in any of the following programs at WFUSM: the “Health Justice Advocacy Certificate Program,” the “Health Equity Certificate Program,” the “Health Policy Student Interest Group,” or membership in the American Medical Association. Table 1 summarizes participant demographics.

## Survey results

### Factors contributing to patient health

**Figure 2.**
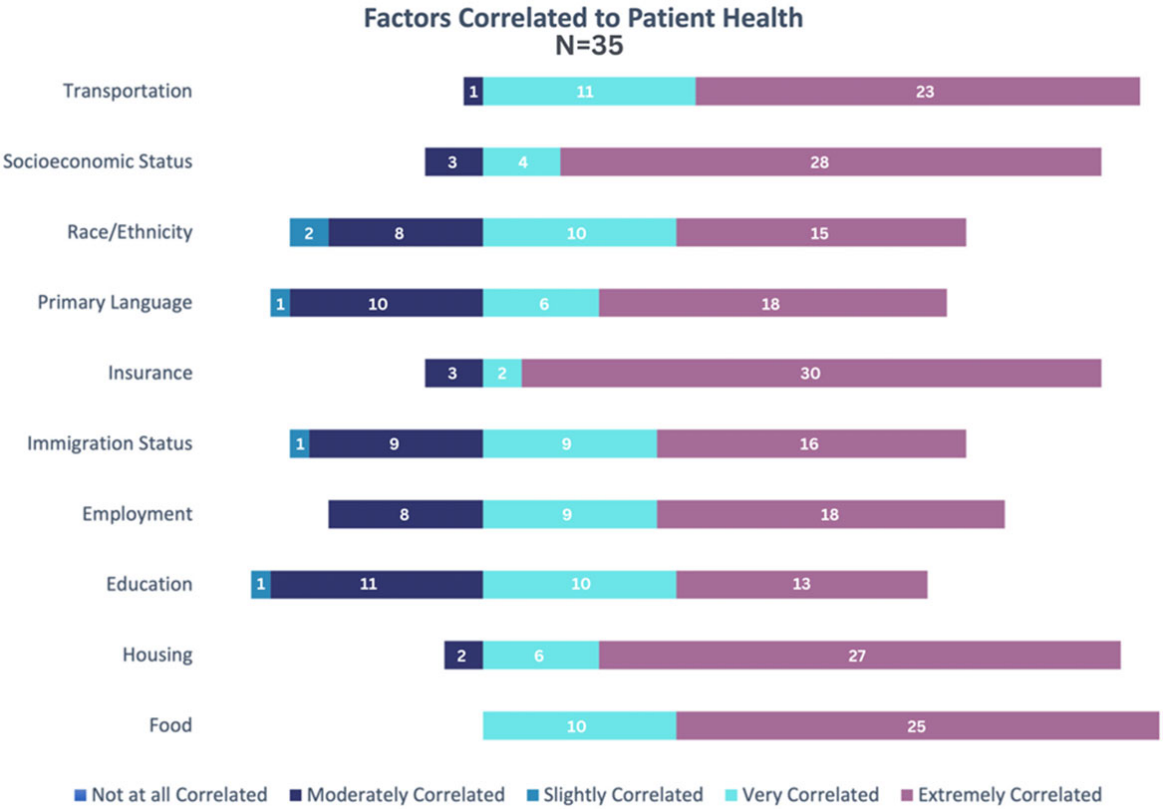
Distribution of survey participant responses on correlation between societal factors and patient health.

When asked to rate the contributions of various SDOH to patient health on a scale from “not at all correlated” to “extremely correlated,” survey participants selected extremely correlated” 60.9% of the time (213/350 total ratings). The majority of participants rated all ten factors either “very correlated” or “extremely correlated” to patient health. The only factor in which all respondents rated the factor as either “very correlated” or “extremely correlated” was access to enough nutritious food. There were five factors (transportation, socioeconomic status, primary language, insurance, and housing) in which the bulk of respondents rated the factor as “extremely correlated.” These outcomes are modeled in Fig. 2.

### Confidence ratings

**Figure 3.**
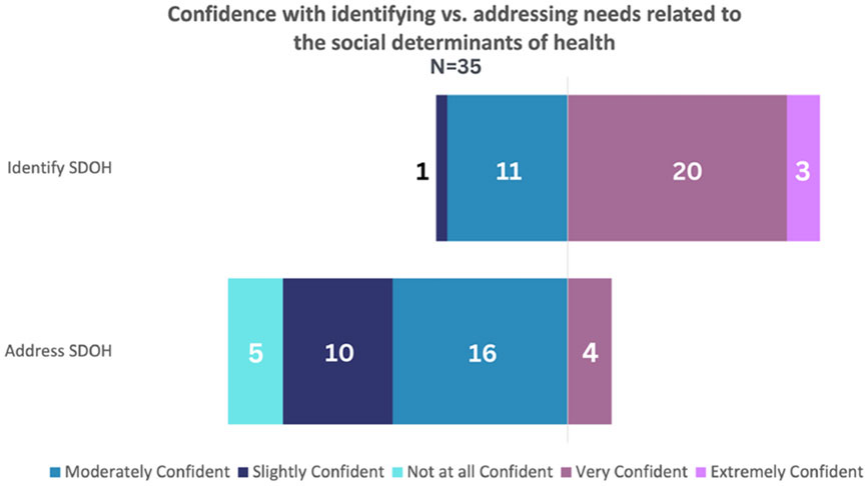
Distribution of survey participant responses on personal confidence in identifying and addressing needs related to social determinants of health.

When asked to rate confidence with identifying SDOH needs in patients, 65.7% (23/35) felt “very confident” or “extremely confident.” This can be contrasted with participants’ feelings of confidence related to addressing these needs in individual patients and communities, in which only 11.4% (4/35) felt “very confident.” No participant reported feeling“extremely confident” with their ability to address SDOH needs. Confidence related to SDOH outcomes is modeled in Fig. 3.

## Qualitative themes describing participant perspectives from interviews

We identified six themes from the interviews: (1) participants recognized that involvement in health policy and/or advocacy is a duty of physicians; (2) participants acknowledged physicians’ voices as well respected; (3) participants were comfortable identifying social determinants of health but felt unprepared to address needs; (4) barriers to future involvement included intimidation, self-doubt, and skepticism of impact; (5) past exposures and awareness of advocacy topics motivated participants to engage in health policy and/or advocacy during medical school; and (6) participants identified areas where the training on these topics excelled and offered recommendations for improvement, including simulation, earlier integration, and teaching on health-related laws and policies. Below we provide representative quotes in support of these themes. These quotes were selected from the broader narrative data and illustrate each theme but do not encompass the entire data set. Additional representative quotes for each theme are included in Table 2.

**Table 2.** Themes and additional representative quotes

Theme	Representative quote(s)
**Participants recognized that involvement in health policy and/or advocacy is a duty of physicians.**	“Health advocacy is important while you’re working, but I feel like it can go beyond the walls of the hospital. Whether it be talking to politicians, sending messages, or just promoting widespread knowledge of available resources to the community…I feel like that’s an important part of being a doctor [and] being a medical student.”
	“I think that we should have the general awareness of how law and medicine interact and what our role is in that. And, you know, not necessarily what our role is in that, but that there is a role and then each individual can decide how they want that role play out in their career.”
***Participants acknowledged* *physicians’ voices aswell-respected.***	“Once you’re a doctor, you kind of have more say and more pull and…a patient complaining about insurance probably isn’t going to do much. But I think if enough doctors agree upon it, and they all work towards it, then it can really make a change.”
***Participants were comfortable identifying SDOH but felt unprepared to address needs.***	“You learn so much about these social determinants of health like transportation, medication affordability, and insurance, and all this stuff, and then no one tells you what you’re supposed to do about it”
	“It’s something you do not really think about when you’re like “oh I’mgoing into medicine” like…you think it’s just gonna be like a learn all the stuff that can go wrong and how to help it. But Wake’s done a good job of like [showing us] all the different things that can be going on and how they impact health.”
	“They give us plenty of information on how to talk to the patient and deal with that, but outside of that…the kind of what to do next, I’m still pretty unsure.”
***Barriersto future involvementincludedintimidation, self-doubt, and *skepticism* of impact.***	“If you’re really gonna get something done, you have to have close-hand experience and then be experienced in whatever field you’re working on, and I think as a first-year medical student, it’s not as convincing as a seasoned physician.”
	“I do not know, there’s a lot of like helplessness I feel like that develops from preclinical education to clinical education where it’s like “Oh yeah like in an ideal world like we could get them food or transportation or refer them to a specialist.” But, in reality, they cannot pay for those things and because people have to set boundaries, I cannot reasonably give everybody who asks a ride or a dollar, so it’s really tough”
***Past exposures and awareness of health policy and/or advocacy topics motivated students to engage in advocacy during medical school.***	“I was a health policy minor in college, so I did take administrative classes and health econ[omics], and I’ve just found that that has been very useful to me. I know what Medicare is; I know what Medicaid is.”
	“I’ve seen issues that I was frustrated when they weren’t addressed. And, um, and then instead of just like being frustrated, you know, like it’s better to try and do something than to sit there and watch it happen if watching it happen bothers you. Which I think most people if they actually see it happening, it bothers them, right?”
***Participants identified areas where the training on these topics excelled and offered recommendations for improvement, including simulations, earlier integration, and teaching on health-related laws and policies***.	“I definitely think first and second year[s] could afford a few classes on just basic like concepts of who we’re treating, what we’re treating, what [the] basic concerns of the population are going to be”
	“If you have an experience where you can actually talk to a social worker in the hospital and make a WIC (Women, Infants, and Children) referral or go through the social determinants screenings in the outpatient setting, it can like make it make a little bit more sense.”
	“MAPS (Medicine and Patients in Society) really did open my eyes, and I was like “wow OK.” So now whenever I see a patient, I like obviously am thinking like what could be going wrong, but also it’s like what could be happening in their life that is detrimental to whatever the situation is. I think without that training, I probably just wouldn’t even notice it or really think about it too much unless it was like glaringly obvious, but a lot of the times I do not think it is.”
	“I feel like MAPS is kind of weird because it’s like, “this is this problem,” and then we discuss how we feel about it, but then we do not do anything about it. So I feel like it’ll be better like [as a] service learning type thing.”

### Participants recognized that involvement in health policy and/or advocacy is a duty of physicians

All participants agreed that there is an inherent responsibility within the medical profession to serve as advocates in some capacity, whether that be individually, at the community level, or nationally. Participants acknowledged that advocacy could look many different ways depending on the scale of the work being done.

*“Advocacy can look like a lot of different things. On one hand there’s policy advocacy…on the other hand there’s social advocacy, trying to communicate to the general population issues that are important and increase understanding or affect opinions about different issues. And then there’s also a very small scale, like in personal interactions.”*

However, while acknowledging health policy and/or advocacy as a duty of the profession, some students noted that not all physicians must be engaged in advocacy work to the same extent, with some finding it more of a calling than others.

*“There have been times during medical school where I feel like “this is just a job. I’m just gonna go in and be very good to my patients, and I will go home,” which is very valid because we do think of medicine as a calling, but in the long run we have to…take care of ourselves before we can dedicate our entire lives to the…greater cause.”*

### Participants acknowledged physicians’ voices as well-respected

Every interview participant agreed that carrying the MD degree designation increases physicians’ potential impact. Many students found this motivating, looking ahead to future advocacy work, while others shared a reluctance to engage in advocacy work, citing feeling less influential or more unsure of their perspectives as students without this same status or experience.

*“I think in medicine in our society, physicians do have some power and privilege, in that what we say is taken very seriously.”*

*“I’m still a student, and I do not feel like someone who has that much sway quite yet.”*

### Participants were comfortable identifying SDOH but felt unprepared to address needs

All participants felt comfortable identifying equity concerns related to SDOH while interviewing patients or during simulated scenarios. This stood out as a success from the WFUSM health advocacy and health equity curriculum, with the majority of participants reporting satisfaction. Students emphasized simulated or real patient encounters, rather than didactic lectures, as an effective way to practice identifying and discussing SDOH issues.

*“But the Wake Forest curriculum has been great in showing you…there are health care issues in the community and disparities within the community of people coming to get health care…at the hospital.”*

*“I would not say during lectures, but I think seeing the [standardized patients] and also just seeing the patients in the hospital, you kind of pick it up. Some of those simulated encounters where it’s not just cut and dry…really help. And you get a good feel and it’s a good place to practice because if you mess up there’s nothing on the line.”*

Although participants felt they were able to confidently identify issues related to SDOH as a result of successful training, many students were uncertain about the next steps upon ascertaining this information from patients. Most participants felt this to be evidence of shortcomings in the WFUSM advocacy training and felt guilty about their inability to remedy situations after identifying them and discussing them with patients or standardized patients.

*“We learn a decent amount about social determinants of health, and we learn about some of these barriers to care. We do not learn a ton about how to actually address them and tangible ways that we can connect patients to resources.”*

### Barriers to future involvement included intimidation, self-doubt, and *skepticism* of impact

Though every participant shared the belief that health policy and/or advocacy is central to medicine, few felt comfortable establishing themselves as advocates in practice due to limited opportunities prior to clinical rotations, the existing power differential in academic medicine, and differences related to level of training.

*“A lot of times as a third-year student we’re just trying to fit in. Being involved in advocacy can bring a big spotlight on you depending on how other people…view certain issues. At times it can be a little intimidating.”*

### Past exposures and awareness of health policy and/or advocacy topics motivated students to engage in advocacy during medical school

The minority of participants who felt comfortable engaging in health policy and/or advocacy were involved in focused advocacy training at WFUSM or had second degrees, such as a Master of Public Health or Juris Doctor contributing to their knowledge base and confidence. Participants with this education background often cited this background as motivational and foundational to their ability to speak on advocacy topics in medical school.

*“During my gap year, I had a few patients where the social determinants of health were very ingrained, and I can see that person’s story. I think that’s where you become more passionate about things…when you’re like “this person really impacted my life,” not necessarily by the health condition they had, but it’s everything else that came with them.”*

Despite the existing epidemiology and ethics topics embedded within the pre-clerkship curriculum at WFUSM, many participants felt that the scant exposure to health policy and advocacy subjects or training in the first two years of medical school hindered their development of advocacy skills. This was mainly due to a lack of awareness about the local patient population, their specific healthcare needs, and their barriers to accessing healthcare.

*“I was talking to a lot of students who didn’t realize what the catchment area of our hospital is, and it’s quite wide and varied. So, I think even just having some kind of population health could be good for first and second year, [to show] “these are who our patients are, these are some of their backgrounds.””*

### Participants identified areas where the training on these topics excelled and offered recommendations for improvement, including simulations, earlier integration, and teaching on health-related laws and policies

Participants in their clerkship years universally regarded the health equity curriculum that is embedded in each clerkship as highly beneficial in familiarizing students with issues facing members of our communities and exposing students to some resources that exist within the community.

*“I think throughout third year what I appreciated is the health equity thread that I think every rotation has. It’s more so to teach about how social determinants of health can show up in the clinical setting and less more so how to actively deal with that and advocate, but I do think it’s still good to keep that as a thread throughout to be more aware.”*

Participants often found the “Health Equity Thread” during their clerkships so helpful that they encouraged earlier integration of the content in the pre-clinical phase when many students are doing a significant portion of their community service.

*“This thread that they have in 3rd year is trying to address [a deeper knowledge of things you can advocate for with your patients] as it’s up and coming. I think the doctors that are in charge ofthatare really motivated. I just honestly wish that thread was a little earlier in the curriculum.”*

Each student was able to identify changes to better the existing curriculum or add missing components. Across the board, participants felt that more “real-life” experiences, such as simulated patient encounters, earlier on in training would provide increased exposure and confidence that many felt they lacked.

*“Some of those simulated encounters where it’s not just cut and dry…they really help. And it’s a good place to practice because if you mess up there’s nothing on the line. So it’s a good place to try out what you might do and then tweak it from there if you do not feel comfortable”*

Additionally, most participants agreed that enhanced training specifically related to policy and health insurance would fill a notable gap in their education and improve their ability to care for patients.

*“Learning about insurance early on in medical education would be something that would be super important because that’s how a lot of decisions are made. “What is Medicare, what is Medicaid, what’s a private payer insurance, what’s a PPO, what’s an HMO?’ All of these are just brushed upon.”*

## Discussion

Advocacy has long been intertwined with the role of physicians, from Dr. Rudolf Virchow’s pioneering work on SDOH to its modern inclusion in HSS, now more formally considered the third pillar of medical education alongside basic and clinical sciences. This single-institution study explored medical students’ perceptions and experiences with health policy and advocacy training and practice, and the motivations and barriers for engagement in these areas. Participants recognized the importance of pursuing health policy and advocacy as physicians but noted barriers to future involvement. They expressed appreciation for their training in these areas, particularly for its exposure to the SDOH but felt unprepared to address them. Participants reporting consistent involvement in advocacy efforts often had backgrounds in public health or health policy or significant personal experiences. All participants were able to identify aspects of the WFUSM curriculum that allowed them to better identify issues related to health policy, advocacy, and the SDOH, but many suggested earlier integration of the experiential course from the clerkship year to help with building confidence in practice.

From this study, interview participants unanimously acknowledged advocacy as an inherent duty of physicians, emphasizing the belief that physicians bear a responsibility to advocate for both individual patients and broader communities. This stance resonates with the ethical imperative for physicians to champion the well-being of their patients outside the confines of medical facilities. Such sentiments have been well-documented in medical education research. For instance, a cross-sectional study conducted by Chimonas *et al.* in 2021 revealed that medical students exhibit a clear interest in civic engagement and firmly believe that physicians should actively participate in advocacy efforts [19]. However, within our study, it is important to note that participants recognized the potential variability in the extent of engagement in advocacy and health policy among physicians, with some feeling a stronger inclination than others. This observation mirrors findings from a qualitative study by Griffiths et al., which reported divergent levels of interest in advocacy engagement beyond hospital walls, with a notable portion of students expressing hesitancy regarding the inclusion of physician advocacy as a core competency in UME [30].

The study revealed that medical students recognize the significant influence of social factors on overall health. This understanding is evident from their high ratings of the correlation between these factors on the survey. These findings align with existing literature, which consistently emphasizes the widely held belief in a strong connection between patient health and various factors including insurance status and nutrition [19]. The well-established and widely acknowledged relationship between overall patient health and SDOH highlights the importance of integrating SDOH-focused interventions into healthcare delivery. It further supports the notion that physicians should be knowledgeable and proactive in addressing these needs when considering overall wellness of their patient population.

The integration of health policy, advocacy, and SDOH education in UME is an ongoing topic, with discussions focusing on optimal teaching methods. A commonly presented framework, the 5As framework, established by the National Academies of Sciences, Engineering, and Medicine, offers five avenues to address SDOH: awareness, adjustment, assistance, alignment, and advocacy [31–33]. Within the curriculum at WFUSM, advocacy training occurs in both pre-clerkship and clerkship settings. While the Population Health course and the Health Equity Thread expose students to local community challenges, some participants felt these initiatives didn’t adequately boost confidence in applying knowledge beyond simulated patient encounters. This aligns with survey findings indicating less confidence in addressing SDOH compared to identifying related issues in patient interactions. Interviewees were comfortable identifying SDOH but felt ill-equipped to address them effectively, highlighting an education gap and the need for ongoing curriculum enhancements. Recent studies show positive impacts of SDOH curricula on student understanding and confidence [11,12]. Recommendations from our study, such as increased exposure through simulations and early integration of advocacy and population health topics, provide valuable insights for curriculum development. These enhancements aim to better prepare students for active engagement with communities beyond identifying challenges.

Participants in our study frequently identified barriers to their involvement in advocacy, including intimidation, self-doubt, and skepticism about their potential impact. Many participants expressed that their limited experience, particularly during the early years of medical school, hindered their confidence and ability to establish themselves as advocates. This finding aligns with existing literature, which often cites a lack of training and time constraints as frequent barriers to consistent advocacy engagement [30,34]. This result highlights the importance of providing more opportunities for students to engage in advocacy early on in their medical education. By doing so, students can gain firsthand experience and build confidence in their advocacy skills. Additionally, participants in our study identified the existing power differential in academic medicine as a barrier, emphasizing the need for a supportive and inclusive environment that encourages student participation in advocacy before becoming a medical resident or attending physician.

The impact of previous exposure and awareness of advocacy topics on students’ motivation to engage in advocacy during medical school cannot be overlooked. Participants with prior focused advocacy training or additional degrees in related fields felt more comfortable and passionate about advocacy work. Exposing students to health policy and advocacy topics early on through seminars, simulations, service learning, and opportunities for independent practice through volunteerism in political advocacy can greatly enhance their readiness and willingness to engage as advocates. Many physicians describe early experiences as drivers for their identity as physician-advocates [35].

Existing literature such as Press et al. demonstrates that mandatory advocacy training, including components like advocacy lectures, self-reflection work, and group community outreach, positively influences the development of advocacy, health policy, and service-oriented mindsets in medical students [36]. Other studies have begun to highlight the impact of focused advocacy training by comparing the attitudes and beliefs of students who receive integrated medical and advocacy training with those who undergo the standard UME curriculum [11,25]. Such studies have begun to shape the future directions for UME curricula and further similar studies will continue to help assess the effectiveness of targeted interventions and identify the most beneficial components of advocacy and health policy education in shaping students’ perspectives and promoting their continued involvement in these efforts.

The study’s limitations must be acknowledged. Firstly, while we were able to reach saturation of themes for the qualitative portion of the study, the modest number of interview participants and small subset representing each year of training limited our ability to compare perspectives. Further, although some findings align with existing literature, the study’s conclusions may not be transferrable as they are drawn from a single institution and represent only the perspectives of students from that institution. Additionally, the demographics of the survey and interview cohorts differed; the interview cohort was predominantly female and represented a more even distribution across different levels of training. It’s important to acknowledge the possibility of a self-selection bias among interview participants, who may have volunteered due to their higher engagement in health policy and advocacy activities. This is evidenced by the majority (11/15, 73%) of interview participants reporting involvement in such efforts. Lastly, the study participants were known to the researcher, so social desirability bias may have affected responses.

## Conclusion

This study highlights the importance of physician involvement in advocacy and health policy among medical students in the US and the need for enhanced education and exposure to prepare these students to address health inequities. It underscores the ethical expectation placed on physicians to advocate for their patients and communities and emphasizes the significant impact of social factors on patient health. This study provides valuable insights into students’ perspectives, motivations, barriers, and recommendations around health policy and advocacy, which can inform curricular improvements and ultimately better equip future physicians to address SDOH and promote health equity.
